# Within and post-trial effects of an intensive lifestyle intervention on kidney disease in adults with overweight or obesity and type 2 diabetes mellitus: a secondary analysis of the Look AHEAD clinical trial

**DOI:** 10.1136/bmjdrc-2024-004079

**Published:** 2024-05-30

**Authors:** William C Knowler, Haiying Chen, Judy L Bahnson, Steven E Kahn, Cora E Lewis, David M Nathan, Robert G Nelson, Scott J Pilla, John P Bantle

**Affiliations:** 1 National Institute of Diabetes and Digestive and Kidney Diseases, Phoenix, Arizona, USA; 2 Department of Biostatistics and Data Science, Wake Forest University School of Medicine, Winston Salem, North Carolina, USA; 3 VA Puget Sound Health Care System and University of Washington, Seattle, Washington, USA; 4 Department of Epidemiology, University of Alabama at Birmingham, Birmingham, Alabama, USA; 5 Massachusetts General Hospital and Harvard Medical School, Boston, Massachusetts, USA; 6 Current: Research Division, Joslin Diabetes Center, Boston, Massachusetts, USA; 7 Department of Medicine, Johns Hopkins University, Baltimore, Maryland, USA; 8 Department of Medicine, University of Minnesota, Minneapolis, Minnesota, USA

**Keywords:** Diabetes Mellitus, Type 2, Kidney Diseases, Life Style

## Abstract

**Introduction:**

The Look AHEAD randomized clinical trial reported that an 8-year intensive lifestyle intervention (ILI) compared with diabetes support and education (DSE) in adults aged 45–76 years with type 2 diabetes and overweight/obesity delayed kidney disease progression. Here, we report long-term post-intervention follow-up for the trial’s secondary outcome of kidney disease.

**Research design and methods:**

We examined effects of ILI (n=2570) versus DSE (n=2575) on decline in estimated glomerular filtration rate (eGFR) to <45 mL/min/1.73 m^2^ or need for kidney replacement therapy (KRT: dialysis or kidney transplant) during intervention and post-intervention follow-up (median 15.6 years overall).

**Results:**

Incidence of eGFR <45 mL/min/1.73 m^2^ was lower in ILI during the intervention (HR=0.80, 95% CI=0.66 to 0.98) but not post-intervention (HR=1.03, 0.86 to 1.23) or overall (HR=0.92, 0.80 to 1.04). There were no significant treatment group differences in KRT. In prespecified subgroup analyses, age×treatment interactions were significant over total follow-up: p=0.001 for eGFR <45 mL/min/1.73 m^2^ and p=0.01 for KRT. The 2205 participants aged >60 years at baseline had benefit in both kidney outcomes during intervention and overall (HR=0.75, 0.62 to 0.90 for eGFR <45 mL/min/1.73 m^2^; HR=0.62, 0.43 to 0.91 for KRT). The absolute treatment effects were greater post-intervention: ILI reduced the rate of eGFR <45 mL/min/1.73 m^2^ by 0.46 and 0.76 cases/100 person-years during and post-intervention, respectively; and reduced KRT by 0.15 and 0.21 cases/100 person-years. The younger participants experienced no such post-intervention benefits.

**Conclusions:**

ILI reduced kidney disease progression during and following the active intervention in persons aged ≥60 years. ILI should be considered for reducing kidney disease incidence in older persons with type 2 diabetes.

**WHAT IS ALREADY KNOWN ON THIS TOPIC**

- In the Look AHEAD randomized clinical trial, an 8-year intensive lifestyle intervention (ILI) compared with diabetes support and education (DSE) in adults aged 45–76 years with type 2 diabetes and overweight/obesity delayed kidney disease progression. Here, we report long-term post-intervention follow-up.

**WHAT THIS STUDY ADDS**

- We examined effects of ILI (n=2570) versus DSE (n=2575) on decline in estimated glomerular filtration rate (eGFR) to <45 mL/min/1.73 m^2^ or need for kidney replacement therapy (KRT) during intervention and post-intervention follow-up (median 15.6 years overall). Incidence of eGFR <45 mL/min/1.73 m^2^ was lower in ILI during the intervention (HR=0.80, 95% CI=0.66 to 0.98) but not post-intervention (HR=1.03, 0.86 to 1.23) or overall (HR=0.92, 0.80 to 1.04). There were no significant treatment group differences in KRT. In subgroup analyses, age×treatment interactions were significant over total follow-up: p=0.001 for eGFR <45 mL/min/1.73 m^2^ and p=0.01 for KRT. The 2205 participants aged >60 years at baseline had benefit in both kidney outcomes during intervention and overall (HR=0.75, 0.62 to 0.90 for eGFR <45 mL/min/1.73 m^2^; HR=0.62, 0.43 to 0.91 for KRT). The younger participants experienced no such post-intervention benefits.

**HOW THIS STUDY MIGHT AFFECT RESEARCH, PRACTICE OR POLICY**

- ILI that started in persons aged ≥60 years reduced kidney disease progression during and following the active intervention. ILI should be considered as one means of reducing kidney disease incidence in type 2 diabetes.

## Introduction

Kidney disease develops in 20–40% of people with diabetes,^1 2^ and diabetic kidney disease (DKD) is the leading cause of end-stage kidney disease in the USA and worldwide.^3 4^ Moreover, DKD substantially increases the risk of cardiovascular mortality.^3 5^ Established interventions that prevent or retard DKD include optimizing glycemic^6–8^ and blood pressure^8 9^ control, inhibiting the renin–angiotensin–aldosterone system,^8 9^ and treatment with sodium-glucose co-transporter 2 inhibitors^8–10^ or glucagon-like peptide 1 receptor agonists.^8 9 11^

Interventions that may retard DKD, but for which there is limited evidence, include restricting dietary protein and sodium intake,^8 9^ and increasing moderate-intensity physical activity.^9^ Weight loss through caloric restriction and increased physical activity may also be beneficial, as shown in the Look AHEAD (Action for Health in Diabetes) clinical trial.^12^

Look AHEAD was a multicenter, randomized clinical trial of an intensive lifestyle intervention (ILI) designed to reduce caloric intake, increase physical activity, and establish weight loss which was compared with a control group who received only diabetes support and education (DSE). ILI did not reduce the incidence of the primary composite cardiovascular endpoint.^13^ In a secondary analysis, however, compared with DSE, ILI reduced by 31% the incidence of very high-risk chronic kidney disease (CKD) during a median of 8 years of study intervention (HR=0.69, 0.55 to 0.87), and it reduced the decline in estimated glomerular filtration rate (eGFR) to <45 mL/min/1.73 m^2^ (HR=0.79, 0.66 to 0.96).^12^ Very high risk was deﬁned as (a) eGFR <30 mL/min per 1.73 m² regardless of urine albumin–creatinine ratio (ACR); (b) eGFR <45 mL/min/1.73 m² and urine ACR ≥30 mg albumin/g creatinine; or (c) eGFR <60 mL/min/1.73 m² and urine ACR >300 mg albumin/g creatinine.^9^ There was also a non-significant reduction in incidence of kidney replacement therapy (KRT) (HR=0.80, 0.49 to 1.30). We now report post-intervention follow-up for decreased eGFR and KRT during a median follow-up of 15.6 years since randomization and evaluate effect modification by baseline variables, including age.

## Methods

The trial is registered at ClinicalTrials.gov (NCT00017953) where a detailed study description can be found. Protocols for the different phases of Look AHEAD are available at https://www.lookaheadtrial.org/public/protocols/LookAHEADTrial.pdf, https://www.lookaheadtrial.org/public/protocols/LA-Continuation.pd and https://www.lookaheadtrial.org/public/protocols/LA-Extension.docx.

### Study design and participants

The study design and primary results of the Look AHEAD trial have been described in detail.^12–15^ Briefly, Look AHEAD included 5145 participants with clinically diagnosed type 2 diabetes at 16 clinical sites in the USA. Participants had a diagnosis of type 2 diabetes by their self-report with verification by review of medical records, glycemic testing, and/or report of their healthcare provider. The Look AHEAD cohort therefore reflects a population of patients who were clinically thought to have type 2 diabetes. It is possible, as in clinical practice, that some participants were misclassified. Given the relatively low prevalence of type 1 and monogenic diabetes, and that Look AHEAD only enrolled adults with overweight or obesity, we suspect these types would be rare. Major eligibility criteria were age 45–76 years, self-reported type 2 diabetes mellitus verified by medical records or glucose-lowering medication use, and body mass index (BMI) ≥25 kg/m^2^ or ≥27 kg/m^2^ if taking insulin. Exclusion criteria included inability to walk two blocks, amputation of a lower limb for non-traumatic causes, urine dipstick protein of 4+ (equivalent to approximately >1 g protein/day), serum creatinine exceeding 1.4 mg/dL in women or 1.5 mg/dL in men, current treatment with dialysis, and history of bariatric surgery. There were no exclusions based on other complications of diabetes. Participants could be using any type of glucose-lowering medicine, but the percentage of participants using insulin was limited to <30%. The trial included participants with and without a history of cardiovascular disease (CVD) which was defined as prior myocardial infarction, stroke, congestive heart failure or interventional procedures for CVD (coronary artery bypass grafting, percutaneous transluminal coronary angioplasty, carotid endarterectomy, angioplasty of a lower extremity artery, or aortic aneurysm repair). All participants had to complete a maximal exercise treadmill test before randomization. Data collected prior to or at the time of randomization are referred to as ‘baseline’.

Race/ethnicity was obtained from self-report by asking participants: ‘Are you Latino, Hispanic or of Spanish origin? If not, which best describes you: (1) African American/black, (2) American Indian/Alaska Native, (3) Asian, (4) Native Hawaiian or Pacific Islander, (5) white, or (6) other (specify).’ Because of small numbers, categories 3, 4 and 6 were combined.

### Interventions

Randomization to ILI or DSE, in a 1:1 ratio, occurred from August 2001 through April 2004. ILI consisted of individual and group behavioral counseling sessions and strategies to achieve and maintain ≥7% weight loss including caloric restriction to 1200–1800 kcal/day and increased physical activity (≥175 min/week of moderate-intensity physical activity) as described in detail elsewhere.^15^ In brief, the physical activity goal was ≥175 min/week of moderately intense activity, achieved by the sixth month. Participants were instructed to engage in brisk walking or similar aerobic activity. The activity program relied on unsupervised (at-home) exercise. In the first month, participants were instructed to walk (or otherwise exercise) for at least 50 min/week. Activity was increased to ≥125 min/week by week 16 and ≥175 min/week by week 26. Group sessions reviewed numerous activity-related topics, including methods of exercising safely, and the benefits of strength training, which may comprise up to 25% of the weekly goal. Participants were also encouraged to increase their lifestyle activity by methods such as using stairs rather than elevators, walking rather than riding, and reducing use of labor-saving devices. Participants used a pedometer to increase their daily steps until they reached a goal ≥10 000 steps/day. Interventions were provided regardless of participants’ ages, with activities limited only by disability or illness. During the post-intervention follow-up, no diet or physical activity training was provided.

The DSE included less frequent group sessions focused on education about nutrition, exercise, and social support. The study did not set goals for control of glycemia or CVD risk factors in either group; these were managed by participants’ healthcare providers outside of Look AHEAD.

The intervention was stopped in September 2012 after a median follow-up of 9.6 years on the recommendation of the Data and Safety Monitoring Board because of futility to detect a treatment effect on the primary outcome, a composite of cardiovascular events.^13^ Thereafter, Look AHEAD continued as an observational follow-up study. Data contributing to the current analyses were collected through June 30, 2020, when follow-up visits ended after median post-intervention and total follow-up of 7.5 years and 15.6 years, respectively.

### Outcomes

Data were obtained by trained, certified staff. Outcomes assessors and laboratory staff were blinded to treatment, but participants and interventionists were not because the intervention was behavioral. Serum creatinine was measured annually through year 4 and approximately every 2 years thereafter. Laboratory tests were performed at the Northwest Lipid, Metabolism and Diabetes Research Laboratory, University of Washington, Seattle, as previously described.^12^ Data on KRT (defined as self-reported dialysis or kidney transplantation or death from kidney failure) were collected every 6 months from interviewer-administered questionnaires and death records. Indications for KRT were not ascertained, and such therapy was counted whether given acutely or chronically. Deaths due to kidney failure were ascertained from medical records and adjudicated by reviewers blinded to treatment assignment. 16 deaths in persons who had not reported KRT were due to kidney failure and were counted as KRT outcomes.

When reporting intervention effects on DKD during the active intervention phase,^12^ we used the 2013 Kidney Disease Improving Global Outcomes (KDIGO) classification that incorporated both eGFR and urine ACR.^9^ We discontinued urine ACR measurements during post-intervention follow-up, so further assessment of kidney function was based only on eGFR and self-report of KRT. The outcome of eGFR <45 mL/min/1.73 m^2^ was selected as in our previous report^12^ and defines GFR that is ‘moderately to severely decreased’ or worse.^9^ It was calculated using the new CKD-Epi equation that did not include race as a factor^16^ (eGFR=142 min(Scr/k,1)^α^×max(Scr/k,1)^−1.200^×0.9938^age^×1.012 (if female) where Scr is serum creatinine in mg/dL, k is 0.7 for females and 0.9 for males, α is −0.241 for females and −0.302 for males, min indicates the minimum of Scr/k or 1, max indicates the maximum of Scr/k or 1). As a sensitivity analysis, we repeated the analyses of eGFR using an older CKD-Epi equation which adjusts for black race.^17^ This older equation had been in use for many years and was used in our previous report.^12^

### Statistical analyses

Incidence rates of each outcome were computed as first events/100 person-years of follow-up, with follow-up censored at the last available visit for participants who remained event-free for that condition. Analyses were stratified by randomized treatment group according to the intention-to-treat principle. The time until the first occurrence after randomization was modeled using Cox proportional hazards regression. Intervention effects for ILI versus DSE were reported as rate differences (RDs) and HRs with 95% CIs. Kaplan-Meier estimates were used to calculate the cumulative incidence of an event over time for both study groups and were compared using log-rank tests. The consistency of intervention effects among seven prespecified subgroups defined by age, sex, race/ethnicity, BMI, history of CVD, insulin use, and diabetes duration was assessed with tests for multiplicative interaction with intervention assignment. Age was stratified by baseline age (<60 or ≥60 years) as in our previous report.^12^ The effects of ILI compared with DSE during intervention, observational post-intervention, and the overall follow-up period were shown as RDs and HRs estimated using Cox regression models with the indicator variable for post-intervention visit as a time-varying predictor. We dichotomized age at 60 years because this report is a follow-up of the previous report which used this cut-off point that is rounded from the mean age of 59 years. As supporting analyses, we also tested the interaction by analyzing age in tertile groups and as a continuous variable. As a sensitivity analysis, we evaluated the intervention effect using non-kidney death as a competing risk.^18^

To provide contextual data to interpret kidney outcomes, the effect of the intervention on DKD risk factors—weight, HbA1c, systolic blood pressure and diastolic blood pressure—was analyzed with linear mixed-effects models. The model included an indicator variable for intervention assignment, follow-up time, and the interaction between the intervention and follow-up time while adjusted for baseline measures. Least square means were plotted to portray the trend over time. The average post-randomization levels of these variables for DSE and ILI were estimated and the differences in average levels of non-occupational physical activity reported on the Paffenbarger questionnaire^19^ were compared using linear contrasts. The use over time of medications blocking the renin–angiotensin system, glucagon-like peptide 1 receptor agonists, sodium-glucose co-transporter 2 inhibitors, and metformin was examined as binary outcomes using generalized estimating equations with compound symmetry variance–covariance matrix. A two-sided p value of <0.05 was considered statistically significant. Because the kidney outcomes were highly correlated with each other and were similarly affected by treatment group assignment, we did not adjust p values for multiple comparisons.

We performed a post-hoc analysis stratifying participants by whether they had achieved the treatment goal of losing ≥7% body weight during the first year. Counting of cases and person-years of follow-up started at the 1-year examination for this analysis.

## Results

The baseline characteristics of the 5145 participants randomized in Look AHEAD have been described.^12^ The mean age was 59 years, 60% were women, 63% were non-Hispanic white, 14% had a history of CVD, 16% were treated with insulin, and 4% had high risk or very high risk of CKD according to KDIGO criteria.^9^ Characteristics are stratified by baseline age in table 1. The older participants were more likely to be white, male, and had a longer duration of diabetes, a higher prevalence of history of CVD, lower eGFR, and more advanced categories of kidney disease. Of the 5145 randomized participants, 33 were excluded from the analysis of decline in eGFR because eGFR was <45 mL/min/1.73 m^2^ or missing at baseline. Of the remaining 5112, some follow-up eGFR data were available on 4996 or 97.7%. Further details, including by treatment group, are shown in the flow chart in online supplemental figure 1. The number of participants analyzed for KRT was slightly higher because none were excluded for low eGFR at baseline (online supplemental figure 2).

10.1136/bmjdrc-2024-004079.supp1 Supplementary data

**Table 1:** Baseline characteristics according to baseline age

	Baseline age <60 years		Baseline age ≥60 years	
	ILI	DSE	ILI	DSE
Number	1480	1450	1090	1125
Sex				
Women	948 (64.1)	938 (64.7)	578 (53.0)	599 (53.2)
Men	532 (35.9)	512 (35.3)	512 (47.0)	526 (46.8)
Age (years)	53.9 (4.1)	54.0 (4.1)	64.9 (4.0)	65.2 (4.0)
Race/ethnic group				
White	855 (57.8)	864 (59.6)	766 (70.3)	767 (68.2)
African American	242 (16.4)	247 (17.0)	158 (14.5)	157 (14.0)
Hispanic	242 (16.4)	205 (14.1)	98 (9.0)	135 (12.0)
American Indian	89 (6.0)	96 (6.6)	41 (3.8)	32 (2.8)
Other or mixed*	51 (3.4)	38 (2.6)	27 (2.5)	34 (3.0)
Duration of diabetes (years)	6.0 (6.0)	6.0 (5.4)	7.8 (7.3)	7.9 (7.4)
History of cardiovascular disease	135 (9.1)	120 (8.3)	230 (21.1)	227 (20.2)
Weight (kg)	102.2 (20.6)	102.8 (19.5)	98.3 (18.1)	98.4 (17.7)
BMI (kg/m^2^)	36.6 (6.3)	36.8 (6.0)	34.9 (5.5)	34.9 (5.2)
Waist circumference (cm)	114.3 (15.2)	114.6 (13.9)	113.0 (13.0)	113.4 (13.1)
HbA1c				
Per cent	7.3 (1.2)	7.4 (1.3)	7.2 (1.0)	7.2 (1.1)
IFCC units (mmol/L)	56 (13.1)	57 (14.2)	55 (10.9)	55 (12.0)
Systolic blood pressure (mm Hg)	126.3 (16.2)	128.2 (16.7)	130.7 (18.2)	131.1 (17.3)
Diastolic blood pressure (mm Hg)	70.9 (9.5)	71.3 (9.5)	68.6 (9.5)	69.2 (9.6)
Urine ACR (mg/g), median	8.6 (5.2–18.2)	8.5 (5.2–17.8)	8.6 (5.2–18.3)	9.4 (5.5–21.1)
Urine ACR ≥300 mg/g	41 (2.8)	29 (2.1)	35 (3.3)	38 (3.5)
Serum creatinine (mg/dL)	0.8 (0.2)	0.8 (0.2)	0.9 (0.2)	0.9 (0.2)
eGFR (mL/min/1.73 m^2^), median	101 (87–107)	102 (88–106)	90 (76–98)	91 (75–98)
eGFR <45 mL/min/1.73 m^2^†	4 (0.3)	3 (0.2)	8 (0.7)	6 (0.5)
Insulin treatment	220 (15.4)	224 (16.0)	165 (15.6)	186 (17.2)
Kidney disease category‡				
Normal or low risk	1196 (82.9)	1163 (82.5)	821 (78.3)	821 (75.9)
Moderate risk	196 (13.6)	213 (15.1)	174 (16.6)	203 (18.8)
High risk	47 (3.3)	30 (2.1)	43 (4.1)	52 (4.8)
Very high risk	4 (0.3)	4 (0.3)	10 (1.0)	5 (0.5)

Statistics are number (%), mean (SD), or median (IQR).

All baseline variables differed significantly (p<0.05) by age except for ACR and insulin treatment.

*Other consists of Asian Americans, Pacific Islanders, those reporting a race other than the investigator-specified categories, or those who did not answer. Participants who reported belonging to more than one race were classed as mixed race.

†Includes those with history of renal replacement therapy regardless of laboratory results.

‡From the Kidney Disease Improving Global Outcomes classification.^9^

ACR, albumin-to-creatinine ratio; BMI, body mass index; DSE, diabetes support and education; eGFR, estimated glomerular filtration rate; IFCC, International Federation of Clinical Chemistry and Laboratory Medicine; ILI, intensive lifestyle intervention.

**Figure 1:**
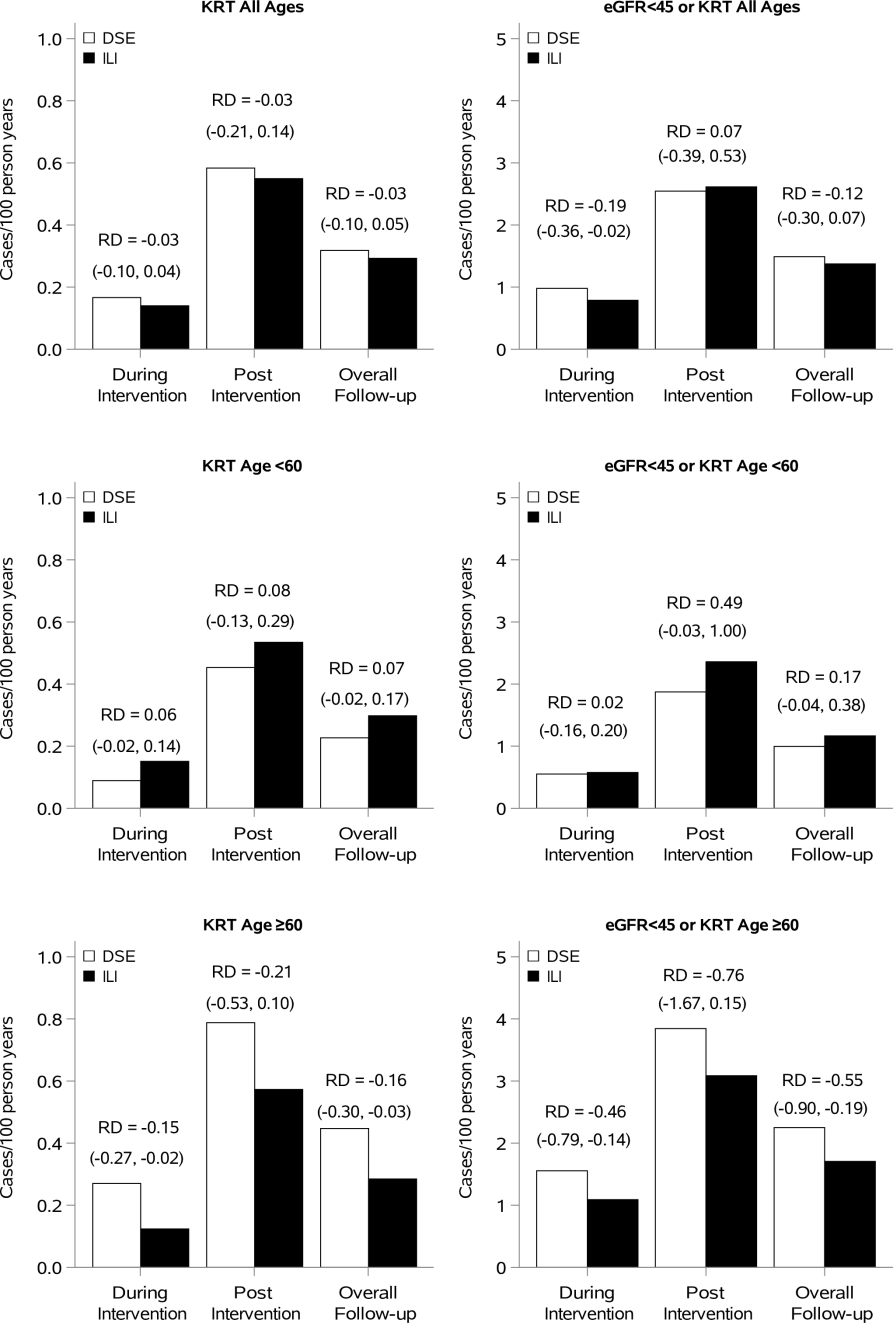
Incidence rates of eGFR <45 mL/min/1.73 m^2^ (left panels), in cases/100 person-years, and kidney replacement therapy (KRT, right panels), by treatment group and study time periods. Treatment effects (ILI vs DSE) are shown as rate differences (RDs) with 95% CIs. Results are shown for all ages (top panels) and in subgroups according to baseline age (<60, middle panels; or ≥60 years, bottom panels). Note differences in scale of vertical axes between the figures for eGFR (left panels) and KRT (right panels). The plotted rates are also shown in online supplemental table 1. DSE, diabetes support and education; eGFR, estimated glomerular filtration rate; ILI, intensive lifestyle intervention.

**Figure 2:**
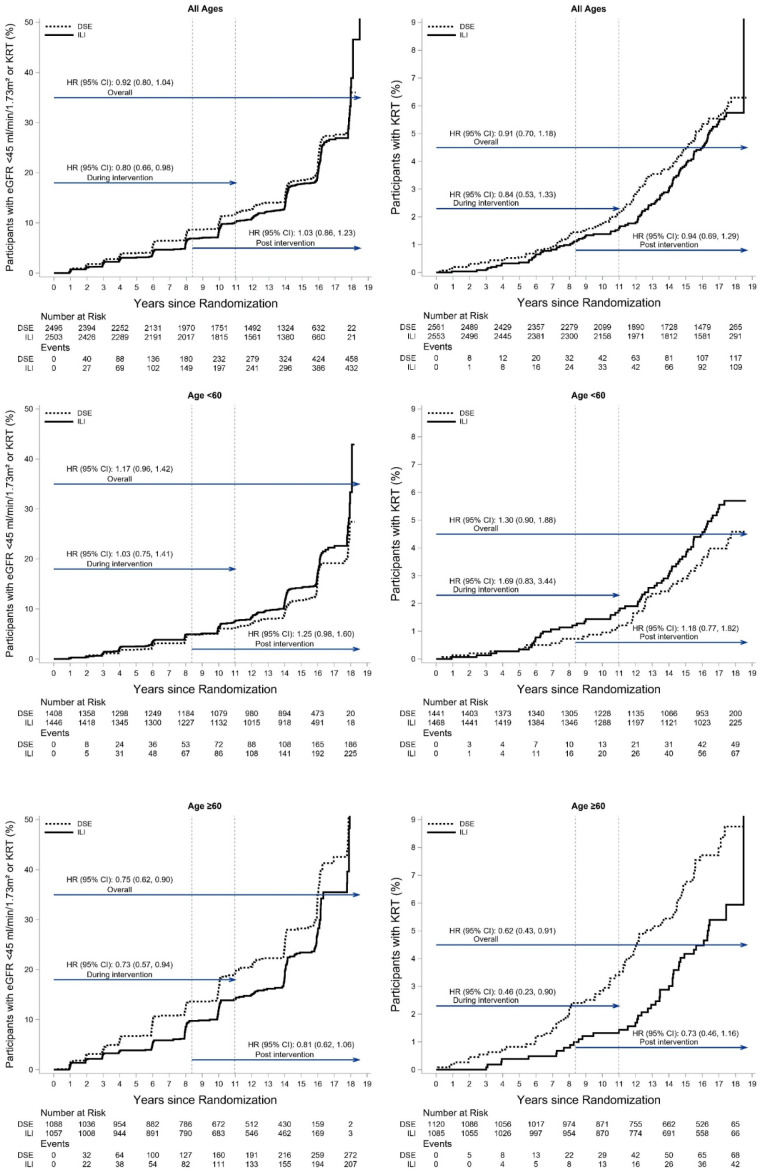
Cumulative incidence of eGFR <45 mL/min/1.73 m^2^ (left panels) and kidney replacement therapy (KRT, right panels) by baseline age during active treatment and follow-up. Results are shown for all ages (top panels) and in subgroups according to baseline age (<60, middle panels; or ≥60 years, bottom panels). Note differences in scale of vertical axes between the figures for eGFR (left panels) and KRT (right panels). The vertical dashed reference lines denote the time period when active intervention ended (a range of times because study enrollment occurred from August 2001 to April 2004, but intervention ended on one date in September 2012 for all participants). The rates for eGFR <45 mL/min/1.73 m^2^ include persons who developed KRT without a study measure of eGFR. DSE, diabetes support and education; eGFR, estimated glomerular filtration rate; ILI, intensive lifestyle intervention.

Incidence rates of eGFR<45 mL/min/1.73 m^2^ and of KRT in the ILI and DSE groups are shown in figure 1 for the entire cohort and stratified by baseline age (<60 or ≥60 years). The results are also shown as cumulative event rates in figure 2. In the post-intervention period, incidence rates of both outcomes shown in figure 1 were higher and, equivalently, the slopes of the cumulative incidence curves in figure 2 were steeper than during intervention, reflecting the greater age and duration of diabetes of the participants during the latter period.

**Figure 3:**
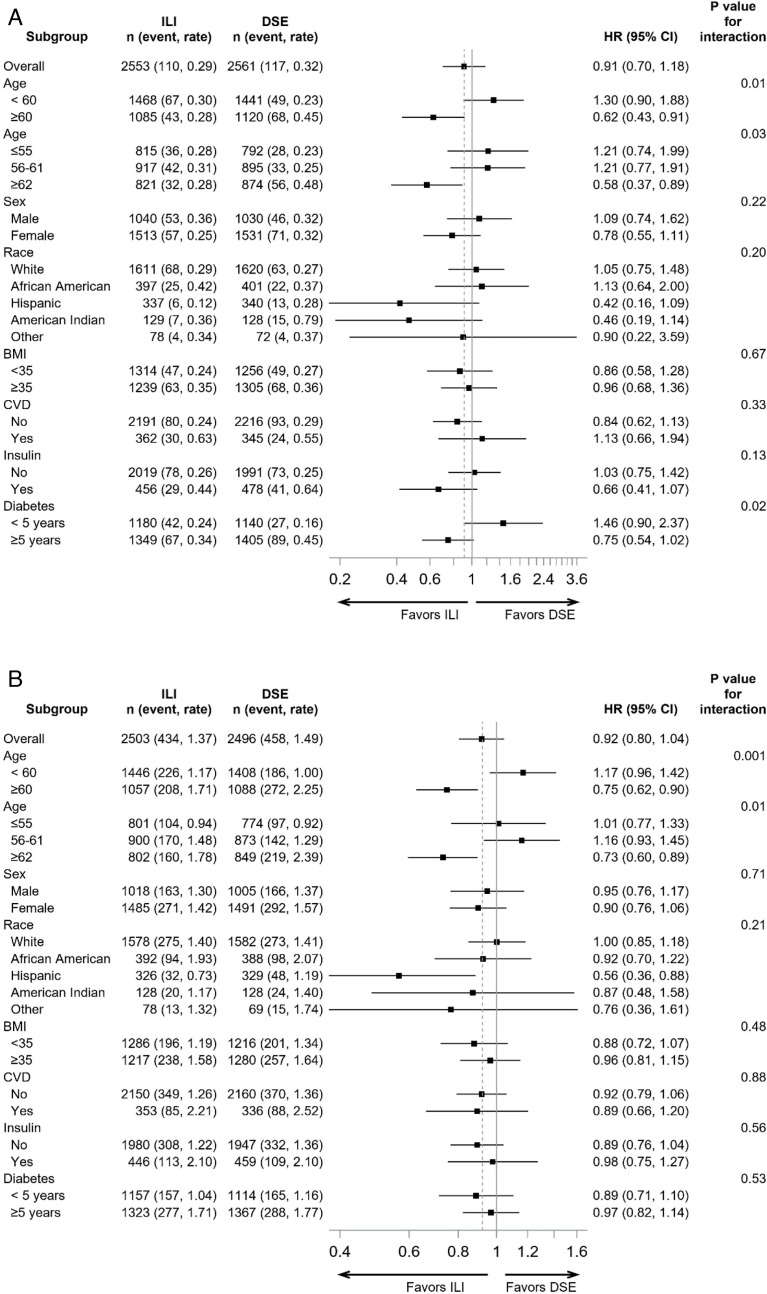
Subgroup analysis of event rates and treatment HRs for eGFR <45 mL/min/1.73 m^2^ (A) and kidney replacement therapy (KRT, B) during the overall study (intervention plus post-intervention). Subgroups are based on baseline characteristics. The vertical dashed reference line refers to the overall HR; n=number of participants in each subgroup, event=number of events in each subgroup, rate=events/100 person-years, HR (rate in ILI/rate in DSE). The rates for eGFR <45 mL/min/1.73 m^2^ include persons who developed KRT without a study measure of eGFR. CVD, cardiovascular disease; DSE, diabetes support and education; eGFR, estimated glomerular filtration rate; ILI, intensive lifestyle intervention.

Subgroup by treatment interaction tests and treatment effects within subgroups for decline in eGFR are shown in figure 3A, for the total follow-up period. There was a significant treatment interaction with age stratified by <60 or ≥60 years at baseline (p=0.001). The treatment by age interactions were also analyzed in tertile groups (45–55, 56–61, and 62–76 years, p-interaction=0.01) and as a continuous variable (p-interaction=0.01). In the subgroup with baseline age ≥60 years, there was a significantly lower incidence of eGFR <45 mL/min/1.73 m^2^ with ILI during the overall study period (HR=0.75, 0.62 to 0.90) that did not apply in the younger subgroup (HR=1.17, 0.96 to 1.42). In those ≥60 years old at baseline, the absolute ILI treatment effect was greater post-intervention than during the intervention (0.46 and 0.76 cases/100 person-years during and after the intervention) as shown in figure 1. Although there was no significant overall interaction by race/ethnicity, ILI appeared to reduce the risk of eGFR <45 mL/min/1.73 m^2^ in Hispanic participants when analyzed separately (HR=0.56, 0.36 to 0.88; figure 3A).

For incidence of KRT, baseline age (<60 or ≥60 years) had a significant treatment interaction (p=0.01, figure 3B). The treatment by age interactions were also analyzed in tertile groups (p-interaction=0.03) and as a continuous variable (p-interaction=0.06). In those aged ≥60 years at randomization, there was a significantly lower incidence of KRT with ILI during the overall study period (intervention plus post-intervention, HR=0.62, 0.43 to 0.91). In terms of absolute incidence rates, in those aged ≥60 years, the ILI decreased KRT by 0.16 cases/100 person years overall (95% CI −0.30 to –0.03). This reduction was smaller during the intervention (0.15 cases/100 person-years) than in the post-intervention period (0.21 cases/100 person-years; figure 1). In the subgroup <60 years at randomization, there was no association between treatment assignment and KRT overall (HR=1.30, 0.90 to 1.88) or during the intervention or post-intervention periods separately. There was also a treatment interaction with diabetes duration (p=0.02) such that ILI appeared to be harmful in those with <5 years’ duration at baseline but beneficial in those with longer duration, although neither of these subgroup associations was statistically significant (figure 3B).

During the intervention, ILI reduced body weight, HbA1c, and blood pressure and increased self-reported physical activity compared with DSE (table 2). Greater reduction in weight in the ILI group persisted post-intervention and overall but the differences between ILI and DSE were smaller. The significant differences between groups in HbA1c and self-reported physical activity disappeared in the post-intervention period but remained significant overall. Lower systolic blood pressure in the ILI group during intervention was replaced by slightly higher systolic and diastolic blood pressure in ILI post-intervention (table 2 and online supplemental figure 3). Use of renin–angiotensin system blocking medicines, glucagon-like peptide 1 receptor agonists, and metformin was less common in the ILI group both during intervention and overall. Sodium-glucose co-transporter 2 inhibitor medications were only used during post-intervention follow-up by a few participants in each group. These findings were similar in those aged <60 or ≥60 years at baseline.

**Table 2:** Interval values for weight, HbA1c, blood pressure, self-reported physical activity, and use of renin–angiotensin system (RAS) blocking, glucagon-like peptide 1 (GLP-1) receptor agonist and sodium-glucose co-transporter 2 (SGLT2) inhibitor medications, and metformin during intervention, post-intervention and overall

Baseline age (years)	During intervention			Post-intervention			Overall		
	ILI	DSE	P value	ILI	DSE	P value	ILI	DSE	P value
All									
Weight (kg)*	94.7±0.2	98.8±0.2	<0.01	92.8±0.2	94.5±0.2	<0.01	93.8±0.2	96.6±0.2	<0.01
HbA1c (%)*	7.01±0.02	7.22±0.02	<0.01	7.45±0.02	7.47±0.02	0.46	7.23±0.02	7.35±0.02	<0.01
Systolic blood pressure (mm Hg)*	124.1±0.2	125.7±0.2	<0.01	130.1±0.3	129.3±0.3	0.04	127.1±0.2	127.5±0.2	0.26
Diastolic blood pressure (mm Hg)*	66.6±0.1	66.6±0.1	0.93	66.8±0.1	65.8±0.1	<0.01	66.7±0.1	66.2±0.1	<0.01
Physical activity (kcal/week)*†	1317.7±26.8	892.0±26.9	<0.01	647.6±30.7	589.2±30.8	0.18	982.6±24.6	740.6±24.7	<0.01
RAS blocking meds (% of participants)‡	70.8 (69.4, 72.2)	75.5 (74.3, 76.8)	<0.01	77.4 (75.6, 79.1)	79.3 (77.6, 80.9)	0.12	74.2 (72.9, 75.6)	77.5 (76.2, 78.7)	<0.01
GLP-1 meds (% of participants)‡§	5.8 (5.2, 6.5)	7.8 (7.1, 8.6)	<0.01	10.7 (9.6, 11.8)	11.9 (10.8, 13.2)	0.12	7.9 (7.2, 8.7)	9.7 (8.9, 10.5)	<0.01
SGLT2 meds (% of participants)‡¶	–		–	3.5 (3.0, 4.1)	3.3 (2.8, 3.8)	0.50	–	–	–
Metformin (% of participants)‡	62.4 (60.9, 63.9)	67.7 (66.3, 69.1)	<0.01	61.7 (59.6, 63.8)	63.2 (61.0, 65.3)	0.35	62.1 (60.5, 63.6)	65.5 (63.9, 67.0)	<0.01
<60									
Weight (kg)*	96.9±0.2	100.4±0.2	<0.01	94.8±0.2	96.3±0.2	<0.01	95.9±0.2	98.3±0.2	<0.01
HbA1c (%)*	7.18±0.03	7.38±0.03	<0.01	7.58±0.03	7.63±0.03	0.18	7.38±0.03	7.51±0.03	<0.01
Systolic blood pressure (mm Hg)*	123.4±0.3	124.6±0.3	0.01	129.3±0.3	128.4±0.4	0.09	126.3±0.3	126.5±0.3	0.67
Diastolic blood pressure (mm Hg)*	67.7±0.2	67.6±0.2	0.72	67.5±0.2	66.8±0.2	0.01	67.6±0.2	67.2±0.2	0.10
Physical activity (kcal/week)*†	1290.7±35.6	893.9±36.3	<0.01	718.8±39.4	651.3±39.9	0.23	1004.8±32.4	772.6±32.9	<0.01
RAS blocking meds (% of participants)‡	71.6 (69.7, 73.4)	74.6 (72.8, 76.3)	0.02	79.8 (77.7, 81.8)	81.0 (78.9, 83.0)	0.41	75.9 (74.2, 77.6)	78.0 (76.3, 79.6)	0.09
GLP-1 meds (% of participants)‡§	6.8 (6.0, 7.7)	9.2 (8.2, 10.4)	<0.01	13.6 (12.1, 15.2)	15.2 (13.5, 16.9)	0.17	9.7 (8.7, 10.7)	11.9 (10.8, 13.1)	<0.01
SGLT2 meds (% of participants)‡¶	–	–	–	4.2 (3.5, 5.0)	4.2 (3.5, 5.0)	0.94	–	–	–
Metformin (% of participants)‡	66.1 (64.2, 67.9)	70.6 (68.7, 72.3)	<0.01	65.9 (63.3, 68.4)	67.9 (65.3, 70.4)	0.27	66.0 (64.1, 67.8)	69.3 (67.3, 71.1)	0.02
≥60									
Weight (kg)*	91.7±0.2	96.4±0.2	<0.01	89.9±0.2	92.0±0.2	<0.01	90.8±0.2	94.2±0.2	<0.01
HbA1c (%)*	6.78±0.02	7.01±0.02	<0.01	7.28±0.03	7.25±0.03	0.522	7.03±0.02	7.13±0.02	<0.01
Systolic blood pressure (mm Hg)*	125.1±0.3	127.1±0.3	<0.01	131.2±0.4	130.4±0.4	0.18	128.2±0.4	128.8±0.4	0.24
Diastolic blood pressure (mm Hg)*	65.1±0.2	65.2±0.2	0.56	65.8±0.2	64.5±0.2	<0.01	65.5±0.2	64.8±0.2	0.01
Physical activity (kcal/week)*†	1351.6±40.6	889.8±40.2	<0.01	537.2±49.1	500.1±48.7	0.59	944.4±38.0	695.0±37.6	<0.01
RAS blocking meds (% of participants)‡	69.8 (67.6, 71.8)	76.8 (74.9, 78.6)	<0.01	73.1 (70.0, 75.9)	76.1 (73.0, 78.8)	0.156	71.4 (69.2, 73.6)	76.4 (74.4, 78.4)	<0.01
GLP-1 meds (% of participants)‡§	4.5 (3.7, 5.5)	5.9 (4.9, 7.0)	0.05	6.1 (5.0, 7.6)	7.2 (5.8, 9.0)	0.28	5.3 (4.4, 6.3)	6.5 (5.5, 7.8)	0.09
SGLT2 meds (% of participants)‡¶	–	–	–	2.3 (1.6, 3.3)	1.7 (1.2, 2.5)	0.24	–	–	–
Metformin (% of participants)‡	57.3 (54.9, 59.6)	64.0 (61.6, 66.3)	<0.01	55.6 (52.1, 59.1)	56.3 (52.6, 59.9)	0.80	56.5 (53.9, 58.9)	60.2 (57.5, 62.8)	0.04

*Mean±SE.

†Self-reported non-occupational physical activity from the Paffenbarger questionnaire,^19^ available for a subset of 2402 participants at baseline, year 1 and year 4 and for all participants at year 8 and afterwards.

‡Mean with 95% CIs.

§Available from year 2 onward.

¶Available from year 10 onward.

DSE, diabetes support and education; ILI, intensive lifestyle intervention.

Online supplemental table 2 shows incidence rates stratified by whether participants met the weight loss goal of ≥7% in the first year. Similar patterns were seen in those who did or did not meet this goal; for each outcome (eGFR or KRT), the estimated effect of ILI was detrimental (HR >1) for those with baseline age <60 years and beneficial (HR <1) when baseline age was ≥60 years. The exception was for those <60 years in the DSE group who lost ≥7% of body weight, for whom the numbers were too small for precise estimates. Given the smaller samples after stratification by weight loss, not all of these effects or interactions were statistically significant.

In the sensitivity analysis computing eGFR using the older CKD-Epi formula,^16^ there was a higher incidence of eGFR <45 mL/min/1.73 m^2^ because more events were identified than with the newer equation. Nevertheless, the effect of ILI on eGFR <45 mL/min/1.73 m^2^ using the older equation (HR=0.92, 0.81 to 1.05) was almost identical to the primary analysis computed using the newer eGFR equation (HR=0.92, 0.80 to 1.04). Findings from the competing risk analysis were not substantially different from the primary analysis (not shown).

## Discussion

In this post-intervention follow-up to the Look AHEAD randomized clinical trial, age interacted with the treatment effects such that ILI reduced the incidence of eGFR <45 mL/min/1.73 m^2^ and of KRT in those ≥60 years old at baseline but had no such effects in the younger participants. During the intervention, there were significant ILI treatment benefits on very high-risk CKD by KDIGO criteria and by eGFR <45 mL/min/1.73 m^2^. Although treatment benefits on these laboratory-based outcomes were established in the earlier analysis, the outcome of KRT had not occurred in large enough numbers (36 in DSE and 29 in ILI participants) for precise estimation of a treatment effect, although a benefit was suggested (HR 0.80, 0.49 to 1.30).^12^ Therefore, we report the kidney disease outcomes of decreased eGFR and KRT during post-intervention follow-up. Of note, in our previous report,^12^ eGFR decline was combined with albuminuria to define an outcome of very high-risk CKD according to the KDIGO criteria.^9^ Since we did not measure urine albumin during the post-intervention follow-up period, we defined DKD in this report by eGFR and KRT alone.

We previously suggested that the beneficial effect of ILI on kidney outcomes was partially mediated by intervention-related decreases in weight, HbA1c, and blood pressure.^12^ The importance of weight loss was supported by a secondary subset analysis of the within-trial results; greater time at ≥7% weight loss during the first 4 years of intervention was associated with lower incidence of declining eGFR.^20^ In the present analysis of the complete study group, the age by treatment interaction persisted regardless of the first-year weight loss (online supplemental table 2). The intervention effects on HbA1c and self-reported physical activity were no longer significant, and effects on blood pressure were reversed during post-intervention follow-up (table 2). Moreover, the modest separation in HbA1c levels between ILI and DSE during the active intervention was not sufficient to provide a long-term legacy effect on kidney outcomes as seen in other clinical trials that created greater separations in HbA1c over time.^21 22^ The beneficial effect of ILI on kidney outcomes in Look AHEAD was not due to use of renin–angiotensin system blocking medicines or glucagon-like peptide 1 receptor agonists as they were used by fewer participants in the ILI group than in the DSE group, both during intervention and overall. Similarly, sodium-glucose co-transporter 2 inhibitor medications were not responsible for the beneficial effect of ILI on kidney outcomes as these newer medications were used by only a few participants in each group and only during post-intervention follow-up. There was lower use of metformin in the ILI group than in the DSE group. Whether metformin has a specific effect on kidney function has not been determined given the conflicting, observational evidence for either beneficial^23–27^ or harmful^27–30^ effects.

We performed our analysis by computing eGFR using a new formula that does not adjust for black race.^16^ This change was made in an attempt to remove racial bias in medical diagnoses and treatment. It has been argued that because omitting the adjustment leads to greater inaccuracy in eGFR among self-identified black people, black race should be used until it is replaced by more accurate measurements.^31 32^ Omission of race in prediction equations in general is problematic when it makes prediction less accurate.^33^ Although we report our main results using the new CKD-Epi formula without race adjustment, we conducted a sensitivity analysis using the previous CKD-Epi formula with race adjustment and found no substantive difference in the results. The event rates during intervention reported here differ slightly from our original report^12^ because of the change in the way we calculated eGFR and because of previously unascertained KRT.

Subgroup analysis suggested that fewer people ≥60 years of age at baseline developed KRT with ILI than with DSE even though the entire group treated with ILI did not. However, the entire ILI group experienced less decline to eGFR <45 mL/min/1.73 m^2^ during the intervention. Only those ≥60 years of age at baseline had this benefit during the overall period of observation. The cause or causes of this statistically significant (p<0.001) age by treatment interaction are not known and could not be explained by age differences in treatment effects on HbA1c, weight, BMI, self-reported physical activity, or blood pressure. Nevertheless, a beneficial effect of physical activity on kidney function in adults aged 70 years and older has previously been reported. An ancillary analysis of the Lifestyle Interventions and Independence For Elders (LIFE) Study found that when compared with a health education intervention, a moderate-intensity physical activity and exercise intervention slowed the rate of decline in eGFR and reduced the likelihood of rapidly declining eGFR in sedentary older adults with or without diabetes.^34^

Limitations of our study include not measuring albuminuria post-intervention, so we could not report some of the outcomes reported previously.^12^ As eGFR is a continuous variable, we chose a cut-off point of <45 mL/min/1.73 m^2^ to define one of the outcomes, because this report is a follow-up of our previous report using this definition.^12^ Further, eGFR values below this point define ‘moderately to severely decreased GFR’ or worse kidney function according to KDIGO.^9^ We did not use <60 mL/min/1.73 m^2^, a common definition of abnormal eGFR, because such eGFRs are frequent in older persons without identifiable kidney disease. Because other treatments, such as medicines for hypertension or diabetes, were not randomized, their effects on the kidney outcomes cannot be easily assessed. Adjusting models for these post-randomization factors can lead to erroneous conclusions,^35^ and analyses to explore potential mediators of the treatment effects are beyond the scope of this paper. Therefore, we report only the intention-to-treat analyses.

Some participants may have had causes of CKD unrelated to diabetes for which lifestyle interventions were not effective. We did not ascertain the assigned cause of kidney disease leading to KRT nor the type of KRT. However, cases of non-diabetic kidney disease that are unrelated to lifestyle should be evenly distributed between the two study groups. We counted cases of KRT whether the dialysis was acute or chronic. While acute kidney injury requiring short-term dialysis is not equivalent to end-stage kidney disease, it may identify persons in whom kidney disease was already present prior to the acute injury, a likely explanation of why it often predicts rapid decline in GFR.^36^

Competing risk of death due to CVD and other causes could also affect the results of this study. However, findings from the competing risk analysis did not support this hypothesis. The limited numbers of KRT cases did not support more detailed analyses of the age by treatment interaction, so we used the same age dichotomy at 60 years as in our previous report. The analyses of age in tertile groups indicated benefits on both kidney outcomes in the age group 62–76 years. Thus, the age at which ILI may be beneficial in delaying KRT can be inferred only approximately.

In sum, ILI reduced progression of kidney disease, defined by eGFR <45 mL/min/1.73 m^2^ or KRT, during the active intervention in persons aged ≥60 years at baseline, but not in the younger participants. These benefits of absolute rate reduction persisted after the intervention was stopped. We suggest that ILI be considered as a means of reducing kidney disease in older persons with type 2 diabetes.

## Data Availability

Data are available in a public, open access repository. Data used for this analysis are publicly available at https://repository.niddk.nih.gov/studies/look-ahead/.
